# Supplementary material for: Human In Vitro Models of Epilepsy Using Embryonic and Induced Pluripotent Stem Cells
Source: Cells. 2022 Dec 7;11(24):3957. doi: 10.3390/cells11243957 (PMC9776452; doi:10.3390/cells11243957)
Supplement: Supplementary file 1 [file cells-11-03957-s001.zip › cells-1946586-supplementary.pdf]

Review

Human *In Vitro* Models of Epilepsy Using Embryonic and Induced Pluripotent Stem Cells

Muhammad Shahid Javaid, Tracie Tan, Naomi Dvir, Alison Anderson, Terence J. O'Brien, Patrick Kwan and Ana Antonic-Baker *

Department of Neuroscience, Central Clinical School, Monash University, Melbourne, VIC 3004, Australia
* Correspondence: ana.antonic-baker@monash.edu

Table S1. Data extracted from the included studies to report the progress of the stem cells field in epilepsy research.

**Reference**	**Year**	**Epilepsy phenotype**	**Cells Type**	**Stem cell type**	**The cell population used in the study**	***Gene mutation***	**Main outcomes**
[1]	2013	Dravet syndrome (DS)	Skin Fibroblasts	iPSCs	GABAergic and glutamatergic	*SCN1A*	The patient's iPSCs-derived neurons showed a functional decline which supports previous findings in murine disease models where loss-of-function in GABAergic inhibition appears to be the main driver in epileptogenesis. This suggests that patient-specific cell-based models may serve as a powerful new research platform to study genetic neuronal disorders including epilepsies.
[2]	2016	Dravet syndrome (DS)	Skin Fibroblasts	iPSCs	GABAergic neurons, glutamatergic	*SCN1A*	CRISPR/Cas9 and TALEN genome editing were used to generate *SCN1A* loss-of-function mutations in iPSCs derived neuronal models. It was observed that the mutations influenced the properties of Nav current and Nav activation in Nav1.1-expressing GABAergic neurons. This study reveals the physiological basis of epileptogenesis caused by *SCN1A* loss-of-function mutation.
[3]	2018	Dravet syndrome (DS)	Skin fibroblasts	iPSCs	GABAergic neurons	*SCN1A*	The patient's iPSCs-derived neurons showed electrophysiological impairments seen in the patient. This suggests that iPSCs-derived neuronal models could be a reliable system to reflect the clinical severities of the patient.

[4]	2013	Dravet syndrome (DS)	Skin Fibroblasts	iPSCs	iPSCs and Neurons	*SCN1A*	Forebrain-like pyramidal- and bipolar-shaped neurons were derived using the iPSCs derived from fibroblasts of two patients and three controls. Patients-derived neurons showed increased sodium currents, spontaneous bursting, and hyperexcitability. Authors claimed their study reveals a previously unrecognized cell autonomous epilepsy mechanism of Dravet syndrome.
[5]	2016	Dravet syndrome (DS)	PBMCs	iPSCs	iPSCs and Neurons	*SCN1A*	The study reported the generation of neurons from the PBMCs derived iPSCs from the patient carrying *SCN1A* mutation mosaicism. The findings of the study suggest that *SCN1A* mutation leads to changes in the dopamine system that may contribute to behavioral abnormalities in Dravet Sydrome.
[6]	2014	Dravet syndrome (DS)	Skin fibroblasts	iPSCs	iPSCs and Neurons	*SCN1A*	Two iPS cell lines were generated using human skin fibroblasts and a point mutation was introduced in the *SCN1A* gene. The engineered iPSCs were tested for pluripotency and functional neurons were derived from them. This suggests that disease-causing point mutations can be introduced in normal hiPS cell lines that can be used to generate a human cell model for studying epileptic mechanisms and for drug screening.
[7]	2018	Dravet syndrome (DS)	Skin Fibroblasts	iPSCs	GABAergic and glutamatergic	*SCN1A*	The patient's iPSCs-derived telencephalic inhibitory and excitatory neurons were used to test the antiepileptic mechanism of cannabidiol (CBD). The treatment with CBD increased the activity of inhibitory neurons and decreased the excitability of excitatory neurons without changing the amplitude of sodium currents. The results suggest a cell type-dependent mechanism of action of CBD in Dravet syndrome that is independent of sodium channel activity.
[8]	2020	Dravet syndrome (DS)	Skin Fibroblasts	iPSCs	Generation of isogenic control (CRISPR/Cas9), GABAergic and glutamatergic	*SCN1A K1270T mutation*	Two isogenic pairs of iPSCs with/without the K1270 T *SCN1A* mutation were created and neurons were derived from them. Comparisons within and between dual pairs of iPSC-derived neurons with *SCN1A* mutation revealed that the mutation reduces sodium current density in excitatory and inhibitory neurons while reducing AP firing only in inhibitory neurons. Thus, causing a hyperactive network. This provides a novel platform to investigate the cellular mechanisms underlying a disease phenotype.
[9]	2013	Dravet syndrome (DS)	Skin Fibroblasts	iPSCs	GABAergic and glutamatergic	*SCN1A*	Functional neurons obtained from the patient's iPSCs with *SCN1A* mutation show hyperexcitability and epileptic phenotype. Analyses revealed that the altered firing of glutamatergic neurons is responsible for mild febrile seizures.
[10]	2019	Dravet Syndrome	Skin Fibroblasts	iPSCs	GABAergic cells, embryoid bodies	*SCN1A*	Patient-specific iPSCs with *SCN1A* mutation demostrated pathways of chromatin remodeling and transcriptional changes in Nav1.1 haploinsufficient GABAergic cells and NPCs were reported.

[11]	2019	*SCN2A* loss of function	Commercially available ESCs	hESCs+G 12:M12	GABAergic and glutamatergic	*SCN2A*	This study reported a detailed characterisation of hESCs-derived neurons using the expression of NEUROG1 and NEUROG 2 together. Molecular, cellular, and electrophysiological properties were studied over 60 days after induction. Results suggested that this approach could be used for neuronal modelling to study functional deficits of various genetic backgrounds.
[12]	2018	Benign Familial Infantile Seizure	Skin Fibroblasts	iPSCs	iPSCs and neurons	*PRRT2*	The physiological role of *PPRT2* was investigated in neurons derived from two siblings carrying heterozygous and homozygous c.649dupC mutation. It was observed that the *PRRT2* directly interacted with Nav1.2/Nav1.6 channels and induced a negative shift in the voltage-dependence inactivation and a slowdown in the recovery from inactivation. The findings suggest disturbance in cellular excitability by lack of negative modulation of Na+ channels appears as a key pathogenetic mechanism.
[13]	2020	Tuberous sclerosis complex (TSC)	Skin Fibroblasts	iPSCs	iPSCs and neural progenitor cells (NPCs)	Unknown	Patient's iPSCs-derived NPCs show that both heterozygous and homozygous loss of *TSC1* influence early neurodevelopmental phenotypes, gene expression, and signaling in NPCs compared to the genetically matched WT cells by mTORC1 activation, which is critical to disease pathogenesis of neurodevelopmental disorders such as ASD. This would be useful for large-scale omics and drug screening studies.
[14]	2019	Tuberous sclerosis complex (TSC)	Commercially available iPSCs	iPSCs	iPSCs and Neurons	*TSC2*	High glucose neuronal differentiation media mask the cellular phenotypes so a careful consideration of culturing conditions is needed for the biological relevance of stem cell-derived neuronal models.
[15]	2019	Tuberous sclerosis complex (TSC)	Skin Fibroblasts	iPSCs	iPSCs and Neurons	*TSC2+/-*	Patient specific iPSCs derived neurons with biallelic mutations of *TSC2* showed hyperactivity and transcriptional dysregulation observed in cortical tubers, which were reversed with rapamycin treatment. It demonstrates that the dysregulation of the mTOR contributes to both phenotypes, which may play a role in the development of cortical tubers.
[16]	2016	Tuberous sclerosis complex (TSC)	Commercially available ESCs	hESCs	iPSCs and Neurons	*TSC2*	This study reported that *TSC2* deletion causes gene-dosage-dependent mTORC1 hyperactivity and alterations in the genes and pathways associated with autism. The *TSC2*+/− and *TSC2*−/− human neurons showed stage-specific synaptic and cellular defects. Moreover, it has been reported that the pharmacological inhibition of mTORC1 corrects synaptic defects independently of early neurodevelopment.
[17]	2018	Tuberous sclerosis complex (TSC)	PBMCs	iPSCs	iPSCs, Neural progenitor cells (NPCs), and Neurons	*TSC2*	Patient iPSCs-derived neurons showed that *TSC2* mutation disrupts neuronal development and modestly activates mTORC1 signaling by PI3K/AKT attenuation, potentially contributing to disease pathology.

[18]	2020	Tuberous sclerosis complex (TSC)	Skin Fibroblasts	iPSCs	Neural progenitor cells (NPCs) and Neurons	*TSC2*	The study reported restoration of *TSC2* loss-of-function in ASD patients' iPSCs-derived neurons using ULK1 and AMPK activators. However, the authors reported the limitations of their study as they used the cells from only one patient and there could be differences across different patients' samples and models and thus, needs further validation.
[19]	2017	Angelman syndrome (AS)	Commercially available iPSCs	iPSCs	knocking out UBE3A	*UBE3A*	Patients' iPSCs-derived neurons with *UBE3A* mutation showed impaired resting membrane potential and action potential, reduced synaptic plasticity, and decreased synaptic activity. This was mimicked using CRISPR.Cas9 based silencing of *UBE3A*. Thus, the cell-based model provides a platform to investigate underlying pathogenic mechanisms.
[20]	2017	Miller-Dieker syndrome (MDS), Lissencephaly	Skin Fibroblasts	iPSCs	iPSCs, Cerebral Organoid	*PAFAH1B, YWHAE*	Cerebral organoids derived from the MDS patient's iPSCs showed cell migration defects, mitotic defects in the outer glia, and severe apoptosis of the neuroepithelial stem cells. This highlights the utility of cerebral organoids for modelling human neurological diseases.
[21]	2016	Myoclonus epilepsy associated with ragged-red fibers (MERRF) Syndrome	Skin Fibroblasts	iPSCs	hiPSCs	mtDNA *A8344G*	This study reported a hiPSCs model of MERRF syndrome to evaluate mitochondrial dysfunction, although the underlying mechanism remains unclear. Both, hiPSCs and hiPSCs-derived cardiomyocytes and neural progenitor cells (NPCs) exhibited fragmented mitochondria with impaired function and increase ROS levels. Future studies are needed to define the effects of impaired mitochondria on ROS levels and their relation to the pathogenesis of MERRF syndrome.
[22]	2018	Diencephalic mesencephalic junction dysplasia (DMJD) syndrome	Skin Fibroblasts	iPSCs	iPSCs and Neurons	*PCDH12*	The patient's iPSCs-derived neuronal progenitor cells showed defective neurite growth due to the loss of *PCDH12*. Thus, the authors proposed that the pathogenic variants of *PCDH12* exhibit abnormalities in white matter tracts due to defects in neurite outgrowth. However, further studies are required to precisely understand the role of *PCDH12* in brainstem development and neural circuit formation.
[23]	2019	Diencephalic mesencephalic junction dysplasia (DMJD) syndrome	H9, Commercially available iPSCs (IMR90)	hESCs	MGE organoids (hMGEOs), Generation of hCOs, and endoderm differentiation	*FOXG1*	Human PSCs were engineered to target endogenous protein for precise dosage control in the hiPSCs and the multiple stages of neuronal differentiation. This study revealed that FOXG1 dose dependency affects the cellular constitution of the human brain, with a 30% threshold for the production of MGE neurons and 60% for the development of GABAergic interneurons. Abnormal neuronal differentiation causes various neurological disorders including epilepsy. This model could be used to understand and treat other diseases caused by the abnormal dosage of specific proteins.

[24]	2018	*PCDH19*- Girls Clustering Epilepsy (*PCDH19*-GCE)	Skin Fibroblasts	iPSCs	iPSCs and Neurons	*PCDH19*	This study reported that loss of *PCDH19* function is linked to increased neural stem and progenitor cells (NSPCs) neurogenesis. The authors proposed that the differences in the neuronal cell production from mutant NSPCs and *PCDH19* wildtype within the same individual may lead to abnormalities and asynchronies in the formation of neural networks, which is associated with the epileptic activity. Further studies are needed to reveal the pathogenic effects of *PCDH19* in detail.
[25]	2018	Lissencephaly	Skin Fibroblasts	iPSCs	iPSCs and Neurons	*DCX*	Patients iPSCs derived neurons with an aberrant DCX protein expression show delayed differentiation, impaired migration, and deficient neurite formation. Thus, these cellular models could further be used to investigate the role of DCX in disease progression.
[26]	2017	Focal Cortical Dysplasia (FCD)	Skin Fibroblasts	iPSCs	iPSCs	*n/a*	This study presented the generation of iPSCs from the skin fibroblasts of two FCD patients. Characterisation of the generated cells confirmed their pluripotency.
[27]	2018	Focal Cortical Dysplasia (FCD)	Skin Fibroblasts	iPSCs	iPSCs and Neurons	*HEY1, NOTCH1, HES1, PAX5*	This study reported the generation of neural progenitor cells from the skin fibroblasts of FCD II patients and healthy individuals. Alteration in the gene expression of Notch signaling was observed in the patient-derived cells compared to the control neuronal cells. This altered gene expression may be related to brain formation with dysplasia.
[28]	2019	Focal Cortical Dysplasia (FCD)	Skin Fibroblasts	iPSCs	iPSCs and Neurons	*NEUROD, ASCL1, DCX2, NEUROG2*	This study presented the gene expression analysis of the neurons generated from the iPSCs of FCD IIb patients and healthy controls. Altered gene expression was observed in the patient derived cells relative to control cells. The different expressions were mainly involved in alterations of the expression of receptors and capture sites, coupling of synaptic vesicles with the presynaptic membrane, synaptic exocytosis, timing, abnormal microtubules, regulation of ion channel, and imbalance of the apoptosis process that may contribute to delays in synaptogenesis.
[29]	2020	Focal Cortical Dysplasia (FCD)	Skin Fibroblasts	iPSCs	iPSCs and Neurons	*CIAP–1, CIAP–2, PI3K,4-EBP1*	Using real-time PCR, the study presented the expression of most of the synaptic and ion channel genes. This study suggested that the cells derived from FCD patients may have more sensitivity to stimuli resulting in altered cell survival, apoptosis, migration, and morphological development.
[30]	2020	Focal Cortical Dysplasia (FCD)	Skin Fibroblasts	iPSCs	iPSCs and Neurons	*DEPDC5*	The patient's iPSCs derived neurons exhibited hyperactivation of mTORC1 and enlarged cell somas that were rescued with the inhibition of mTORC1. This study also reported that cell starvation leads to hyper-activation of the mTOR pathway but the exact mechanism is still unclear.

[31]	2020	Unknown	Commercially available iPSCs	iPSCs	Cortical neurons	Unknown	The study reported the structural and functional development of human neurons and neural networks using a lab-on-a-chip device, a Modular Platform for Epilepsy Modelling *In vitro* (MEMO). Seizure-like events can be induced and targeted to specific networks, and thus, MEMO can model focal seizures in human neuronal networks.
[32]	2018	Juvenile neuronal ceroid lipofuscinosis (Batten disease)	Skin Fibroblasts	iPSCs	iPSCs	*CLN3*	CRISPR/Cas9 mediated mutation correction leads to molecular correction at genomic DNA and mRNA levels in the patient iPSCs lines, crucial for disease modelling.
[33]	2014	Duplication of chromosome 15q11-q13.1	Skin Fibroblasts and HUCBCs	iPSCs	Neurons	*UBE3A*	Gene expression analysis of the patient's iPSCs-derived neurons suggests a possible disruption in the transcriptional regulations of genes in the duplicated 15q11-q13.1 region.
[34]	2017	*CHRNA7* epilepsy	Skin Fibroblasts	iPSCs	iPSCs, NPCs	*CHRNA7*	The iPSCs and iPSCs derived NPCs were generated from the patient's heterozygous 15q13.3 deletions and heterozygous 15q13.3 duplications in *CHRNA7* gene. Molecular investigations of both of the mutations revealed decreased calcium flux. This finding aligns with clinical data, which suggests that both individuals (with deletions and duplications of 15q13.3) display neuropsychiatric disease and cognitive deficits.
[35]	2020	Schizophrenia/autism spectrum disorder	Skin Fibroblasts	iPSCs	Cortical spheroids and 2D glutamatergic neurons	*22q11.2 deletion (incl. DGCR8 gene)*	Patients' iPSCs derived neurons and organoids were used to study 22q11.2 deletion in *DGCR8* gene. Transcriptional profiling revealed changes in the genes of neuronal excitability. Moreover, changes in electrophysiology and calcium signaling were observed. This illustrates how stem cell-based models can uncover the phenotypes associated with genetic forms of neuropsychiatric disease.
[36]	2018	*CAMK2*, synaptic dysregulation	Skin Fibroblasts	iPSCs	iPSCs, Neurons	*CAMK2A*	Neurons derived from patient iPSCs carrying CAMK2A mutation displayed synaptic abnormalities, suggest that a recessive germline mutation in *CAMK2A* leads to neurodevelopmental defects in humans. The study suggests that dysfunctional *CAMK2* paralogs could contribute to other neurological diseases.
[37]	2016	Ohtahara or West syndrome	Commercially available iPSCs	iPSCs	Neuron conditional knock out	*STXBP1*	Patients' iPSCs-derived neurons showed that heterozygous disruption of *STXBP1* produces selective and specific abnormalities in synaptic transmission that may responsible for the severe neuronal disease caused by *STXBP1* mutations in patients.
[38]	2016	Ohtahara syndrome	Commercially available iPSCs	iPSCs	Neurons	*STXBP1*	Patient's iPSCs derived neuronal models showed impaired neurite growth and suggested that decreased expression of STXBP1 leads to changes in the expression and localization of syntaxin-1 which may contribute to the phenotype of Ohtahara syndrome.

[39]	2020	Developmental and/or epileptic encephalopathies (DEEs)	Skin Fibroblasts	hESCs, iPSCs	Neurons	*UGP2*	Brain samples, hESCs, and patients' iPSCSs derived neurons were used to asssess the role of *UGP2*. This study showed that this gene plays an important role in human brain development and start-loss mutations (ATG/methonine mutations) in tissue-specific isoforms of essential genes potentially cause more rare genetic diseases.
[40]	2019	Malignant migrating partial seizures of infancy (MMPSI)/Epilepsy of infancy with migrating focal seizures (EIMFS)	Commercially available iPSCs	iPSCs derived neurons	Generation of a KCNT1 P924L allelic variant,	*KCNT1*	Authors claimed this is the first study to report an increase in the sodium-activated potassium channel (KNa1.1) current in patients' iPSCs derived neurons. This study reported an increased potassium current induced hyperexcitability which could be an underlying factor causing seizures.
[41]	2018	West syndrome	Skin Fibroblasts	iPSCs	Cortical neurons	*ST3GAL3*	Patient iPSC- derived cortcal neurons with *ST3AL3* gene mutation showed changes in the sialylation pattern on the surface of neurons affect adhesive interactions during development, which may cause changes in tissue composition and result in the progression of epilepsy.
[42]	2020	Early-infantile epileptic encephalopathy type 13/*SCN8A*-related epilepsy	Skin Fibroblasts	iPSCs	iPSCs	*SCN8A*	Patients' iPSC-derived neurons with *SCN8A* gene mutation showed increased burstiness and abnormal action potential, sensitive to phenytoin and riluzole. Patch-clamp recordings showed suppressed firing with riluzole. Patients were prescribed riluzole and reported a reduction in seizure frequency and months of seizure freedom. Thus, patient-specific neuronal models could be useful platforms to develop precision epilepsy therapies.
[43]	2020	Pretzel Syndrome (2 patients)	Skin Fibroblasts	iPSCs	NPCs, astrocytes, GABAergic and glutamatergic neurons	*STRADA*	Patient specific iPSC-derived neurons with *STRADA* gene mutation were used to assess the role of *STRADA* in modulation of mTOR signaling and found that this gene is a key regulator of cell size, neuronal excitability, and cortical lamination.
[44]	2021	Neonatal epileptic encephalopathy	PBMCs	iPSCs	iPSCs and Neurons	*KCNQ2*	Patient specific iPSC-derived neurons with *KCNQ2* gene mutation showed faster action potential repolarization, function enhancement of K^+ channels, and larger post-burst hyperpolarization. The study suggests that iPSCs-based model is an ideal platform to study *KCNQ2* mutations and targetting altered ion current could be an effective therapeutic strategy.

[45]	2020	Chorea Acanthocytosis	Unknown	NPCs	Midbrain and hindbrain neurons	*VPS13A*	The fragmentation of the dendritic microtubule, reduction in the number of mitochondria and lysosomes, hyperpolarisation, and proximal axonal deterioration were observed in patient iPSC-derived neurons with *VPS13A* gene mutation. In addition, previously approved pharmacological treatments using other models, were ineffective. Thus, this study suggested that treatment of this multifaceted disease might be neuronal subtype specific, and necessitates the development of precision medicine for this ultra-rare disease.
[46]	2020	MELAS (mitochondrial encephalopathy, lactic acidosis, and stroke-like episodes)	Skin Fibroblasts	iPSCs	Excitatory cortical neurons	*MT-TL1*	The authors used hiPSCs to generate neurons with high levels of m.3243A >G heteroplasmy. It has been reported that higher heteroplasmy leads to neuron-specific mitochondrial dysfunction, reduced dendritic complexity, structural and functional impairments, fewer synapses, and reduced neuronal synchronicity and network activity.
[47]	2010	Rett syndrome	Skin Fibroblasts	iPSCs	iPSCs and Neurons	*MECP2*	The patient's iPSCs-derived neurons with *MECP2* gene mutation recapitulate the early stages of RETT syndrome and represent a promising cell-based tool to screen drugs.
[48]	2012	Hanefeld variant of Rett syndrome	Skin Fibroblasts	iPSCs	iPSCs and Neurons	*CDKL5*	The iPSC-derived neurons from patients with *CDKL5* mutations showed abnormal dendritic spines. Thus, suggested that *CDKL5* is critical for the maintenance of normal synaptic contacts.
[49]	2020	Progressive myoclonic epilepsy (PME) of Unverricht-Lndborg type (EPM1)	PBMCs	iPSCs	Cerebral organoids; fused patterned ventral and dorsal organoids	*CSTB (cystatin B)*	This study invastigated the underlying cellular mechanisms involved in the development of Progressive myoclonus epilepsy (PME) of Unverricht–Lundborg type (EPM1). Low levels of functional CSTB, premature differentiation, alteration in progenitor's proliferation, and changes in interneurons migration were observed in patient derived cerebral organoids.
[50]	2020	Neurochondrin	Skin Fibroblasts	iPSCs	Nneurochondrin deficient iPSCs	*NCDN*	The study generated and characterised the human Neurochondrin deficient iPS cell line (KICRi002-A-3).
[51]	2017	Epileptic encephalopathy), CHD2 haploinsufficiency	Commercially available H9 and Hes3-NKX2-1GFP/W hESC	hESCs	Cortical interneurons, MGE-like progenitors, ventral telencephalic-like neuroectoderm	*CHD2*	Cortical interneurons (cINs) modulate the activity of excitatory neurons and several subtypes of cINs differentiate from medial ganglionic eminence (MGE) during fetal development. This study identifies several aspects of gene-regulatory networks underlying human MGE specification and suggested mechanisms by which the MGE transcription factor (NKX2-1) acts with chromatin remodelling to regulate the gene expression for cINs development.

[52]	2016	15q11.2(BP1-2) deletion	Skin Fibroblasts	iPSCs (3 lines - C2, C4, C5)	iPSCs derived CYFIPI knockdown neural progenitor cells (lentiviral shRNA)	*CYFIP1*	This study reports the knockdown of the *CYFIP1* gene in the neurons derived from patients' iPSCs. The knockdown of the *CYFIP1* gene resulted in the dysregulation of one or more disease gene networks. Thus, this model system could be used for high-throughput drug screening.
[53]	2018	Unknown	Embryonic	hESCs	Long-term self-renewing neuroepithelial cells	*adenosine kinase*	Adenosine kinase deficient neuroepithelial cells were differentiated into neurons and astrocytes and they showed an elevated level of adenosine upon excitation. This could be an attractive therapeutic tool for intrinsic "on-demand" delivery of adenosine in epilepsy and other neuronal disorders.
[54]	2015	Unknown	Embryonic stem cells	hESCs	NPCs	Unknown	Valproate disturbs the mitochondrial morphology and function of hESCs derived neuronal progenitor cells. Using patient-derived models, further studies are needed to investigate the toxicity of clinically approved anti-epileptic drugs.
[55]	2016	Unknown	Commercially available H9, H7 and, iPSC2497	hESCs/iPSCs	MGE and CGE interneurons	Unknown	This study reported the use of combinatorial and temporal modulation of dorsoventral and rostrocaudal signaling pathways to generate Medial Ganglionic Eminence (MGE) cells and Caudal Ganglionic Eminence (CGE) cells, which generate interneurons of specific subtypes. The efficient generation of specific neural progenies from hPSCs will be a crucial step to reveal the full potential of hPSCs in disease modelling, regenerative medicines, bioassays, and drug screening.
[56]	2017	Unknown	Commercially available iPSCs	iPSCs	Neurons and Astrocytes	Unknown	Human iPSCs derived neurons form functional networks and respond to convulsants when co-culture with hiPSCs derived astrocytes.
[57]	2013	Unknown	Carcinoma cell line	human testicular embryonic	Neurons	Unknown	This study highlighted genes and measurable phenotypic changes in response to Lithium and Valproic Acid treatment using hPSCs derived neurons and astrocytes. A combination of expression profiling, phenotypic anchoring and GO analysis were used and further pharmacological analyses are needed for further biomarkers identification.
[58]	2019	Unknown	Commercially available iPSCs	hESCs	hESCs	Unknown	Genotoxicity assessment using hESCs revealed that Carbamazepine, Gabapentin, Lamotrigine, Levetiracetam, or Topiramate caused DNA damage.

[59]	2013	Unknown	Commercially available ESCs	hESCs	Forebrain Interneurons	Unknown	The hPSCs were differentiated into MGE-progenitors and then further into differentiated into GABAergic interneurons and showed mature physiological properties after a long time of up to 7 months, mimics endogenous human neural development. MGE derived cortical interneuron deficiencies cause a broad range of neurodevelopmental disorders, thus highlights the importance of this study.
[60]	2019	Unknown	Commercially available iPSCs	iPSCs	NPCs and Neurons	Unknown	This study reported a protocol to generate neural rosettes, forebrain neural progenitor cells (NPCs), and neurons using iPSCs.
[61]	2019	Developmental and epileptic encephalopathies (DEE)	Skin Fibroblasts	iPSCs	iPSCs	*KCNA2*	This paper desrtibes the generation of patient iPSCs carrying *KCNA2* gene mutation. This cell line could be useful for drug discovery and pathological analyses.
[62]	2013	Unknown	Commercially available H9 and hES3-NKX2.1:GFP	hESCs	Neural progenitors	Unknown	Forebrain progenitors were generated form hESCs. These cells are suitable candidates for cell-based therapies aiming to replace damaged or dysfunctional Cortical hippocampal GABAergic interneurons.
[63]	2016	Unknown	Commercially available iPSC	iPSCs	Cerebral cortical neurons	Unknown	The study reported neuronal development and functional maturation of iPSCs derived neurons for over one year. It was reported that 20-30 weeks are required for mature neuronal activity and the importance of long-term MEA measurements for mechanistic analyses and drug screening. However, culture conditions should be standardised to define maturation time.
[64]	2020	Unknown	Commercially available iPSCs	iPSCs derived cortical neurons	Cortical neurons	Unknown	The hiPSCs derived neurons were used to test seizurogenic compounds using three neuronal models. The differences in seizure liability assessment were reported when compared with rat models. Thus, highlights the need to move to human model systems, thereby eliminating the need for interspecies extrapolation. However, standardised culture and testing conditions are needed with an extensive characterisation of cellular models.
[65]	2014	Unknown	Commercially available iPSCs	iPSCs	Dopaminergic and GABAergic neurons	Unknown	This study reported that multivalent Shh bioconjugates can increase the neuronal commitment of PSCs and thus drive an enhanced and efficient derivation of neurons.
[66]	2015	Unknown	Commercially available iPSCs	iPSCs	Ventral forebrain neurons/ GABAergic neurons	Unknown	The study reported the use of hVGAT-mCherry reporter construct to identify and isolate viable GABAergic neurons derived from PSCs. This approach provides a large quantities of patient-specific viable GABAergic neurons that could be used for disease modelling and drug screening.

[67]	2020	Unknown	Commercially available RC17 and HS1001	hESCs	Glial progenitor cells and GABAergic neurons	Unknown	This study generated functional GABAergic neurons using human glia progenitor cells (hGPCs). The generated interneurons showed postsynaptic activity and an expression of subtype specific markers. Thus, the authors claimed that the generation of GABAergic neurons from a renewable cell line hGPC system could provide a foundation for the development of therapies for interneuron pathologies.
[68]	2015	Unknown	Commercially available H9	hESCs	GABAergic neurons, dopaminergic neurons	Unknown	This study reported a protocol for the generation of midbrain dopaminergic neurons and forebrain GABA using mouse and human PSCs.
[69]	2020	Unknown	Commercially available iPSCs	iPSCs	GABAergic interneurons	Unknown	The authors reported novel transcription factors and genetic regulatory elements that may regulate the genes involved in the development of GABAergic interneurons (GINs). They have performed further analyses to study the functional changes in the genes that play a role in GIN maturation. GINs contribute to normal neuronal excitability and network activity.
[70]	2019	Seizures	Commercially available iPSCs	iPSCs (CNS.4U)	Glutamatergic, GABAergic, dopaminergic neurons and astrocytes	Unknown	The hiPSCs derived neurons were used to study the effects of agonists and antagonists of M1 muscarinic receptors. Neuronal excitability and seizure activity was studied by modulation of M1 receptors or inhibition of Kv7 channels. Thus, this cell-based assay could be used characterization of drugs that affect neuronal activity via M1 receptors.
[71]	2004	Unknown	Commercially available ESCs	hESCs	Adk (adenosine kinase) knockout cells	Unknown	The study reported that Adk−/− ES cells constitute a potential source for therapeutic adenosine-releasing grafts as the Adk−/− ES derived glial cells release adenosine of up to 40.1 ± 6.0 ng per 10^5 cells per hour, an amount considered to be sufficient for seizure suppression.
[72]	2016	Unknown	Commercially available iPSCs and H9, H1	hESCs/iPSCs	MGE interneurons	Unknown	The study stated the generation of different neuronal subtypes using hPSCs. It has been reported that signaling regulators and differentiation paradigms can be integrated together to specify distinct neuronal subtypes to study and treat related neurological diseases.
[73]	2020	Unknown	Commercially available hES1, and hiPSC (Disease: hiPSC)	hESCs/iPSCs	Glutamatergic neurons	*presenilin-1 and presenilin-2*	This study developed a novel metod of neuronal differentation and maturaton by combining two methods, the *NGN2* method and miR-9/9*, miR-124, and Bcl-xL genes (BmiRs) based direct differentiation. Enhanced maturation and synapse formation was observed with increased calcium activity and electrical activity. With enhanced maturation, disease phenotypes can be easily detected at early time points therefore, this could be a very useful tool for drug screening and analyzing neuronal pathophysiology.

[74]	2013	Unknown	Commercially available (H3, H9 and iPSC (C72 and SeV6)	hESCs/iPSCs	Neurons	Unknown	The authors reported a highly efficient derivation of human cortical interneurons using NKX2.1::GFP human embryonic stem cell reporter line. This study defines the signals sufficient to model human ventral forebrain development in the *in vitro* setting. The results of the study lay the foundation to study the involvement of cortical interneurons in human disease pathology.
[75]	2017	Unknown	Lung Fibroblast	iPSCs	Glutamatergic neurons	Unknown	This study showed that the combination of three transcription factors, BRN2, MYT1L, and FEZF2 can directly convert human fibroblasts to functional excitatory cortical neurons. The authors claimed their study to be the first to report the direct conversion of human somatic cells into functional excitatory cortical neurons that have the capacity for synaptic integration into the adult human cortex.
[76]	2018	Dravet syndrome (DS)	Skin Fibroblasts	iPSCs	Patient derived iPSCs	*SCN1A*	The iPSCs with *SCN1A* gene mutation were created from the skin fibroblasts of the patient and the pluripotency of the generated cells was confirmed. This cell line could be useful for drug discovery and pathological analyses.
[77]	2018	Dravet syndrome (DS)	Skin Fibroblasts	iPSCs	Patient derived iPSCs	*SCN1A*	The iPSCs were created from the skin fibroblasts of the patient and the pluripotency of the generated cells was confirmed. This cell line could be useful for drug discovery and pathological analyses.
[78]	2017	Autosomal dominant lateral temporal epilepsy (ADLTE)	PBMCs	iPSCs	Patient derived iPSCs	*LGI1*	The iPSCs with *SCN1A* gene mutation were created from the PBMCs of the patient and the pluripotency of the generated cells was confirmed. This cell line could be useful for drug discovery and pathological analyses.
[79]	2018	*KCNA2* epilepsy	Skin Fibroblasts	iPSCs	Patient derived iPSCs	*KCNA2*	The iPSCs with *KCNA2* gene mutation were created from the skin fibroblasts of patients and the pluripotency of the generated cells was confirmed. This cell line could be useful for drug discovery and pathological analyses.
[80]	2011	Rett syndrome, X-linked epileptic encephalopathy	Skin Fibroblasts	iPSCs	Patient derived iPSCs	*CDKL5*	This study reported the generation of iPSCs from a female and a male patient. Female patient derived iPSCs maintained X-chromosome inactivation and produced either mutant or wild-type *CDKL5* allele which could be identified. The study also reported that the iPSCs can be differentiated into neurons for disease modelling and did not perform further assessment assays.
[81]	2020	Dravet syndrome (DS)	Skin Fibroblasts	iPSCs	Patient derived iPSCs	*Y1102X* in *SCN1A*	The iPSCs with *SCN1A* gene mutation were created from the skin fibroblasts of patients and the pluripotency of the generated cells was confirmed. These cell lines could be useful for drug discovery and pathological analyses.
[82]	2019	*GNB5* epilepsy	Skin Fibroblasts	iPSCs	Patient derived iPSCs	*GNB5*	The iPSCs with *GNB5* gene mutation were created from the skin fibroblasts of patients and the pluripotency of the generated cells was confirmed. This cell line could be useful for drug discovery and pathological analyses.

[83]	2020	Epilepsy-aphasia syndrome (Landau-Kleffner syndrome), GRIN2A Epilepsy	PBMCs	iPSCs	Patient derived iPSCs	*GRIN2*	The iPSCs with *GRIN2* gene mutation were created from the PBMCs of the patient and pluripotency of the generated cells was confirmed. This cell line could be useful for drug discovery and pathological analyses.
[84]	2020	MPPH Syndrome, PIK3R2 Epilepsy	PBMCs	iPSCs	Patient derived iPSCs	*PIK3R2*	The iPSCs with *PIK3R2* gene mutation were created from the PBMCs of the patient and the pluripotency of the generated cells was confirmed. This cell line could be useful for drug discovery and pathological analyses.
[85]	2020	Dravet syndrome (DS)	Skin Fibroblasts	iPSCs	Patient derived iPSCs and isogenic iPSCs control	*SCN1A*	The iPSCs with *SCN1A* gene mutation were created from the skin fibroblasts of the patient and the pluripotency of the generated cells was confirmed. This cell line could be useful for drug discovery and pathological analyses.
[86]	2020	Antecedent febrile seizures (PEFS+)	Skin Fibroblasts	iPSCs	Patient derived iPSCs	*SCN1A*	The iPSCs with *SCN1A* gene mutation were created from the skin fibroblasts of the patient and the pluripotency of the generated cells was confirmed. This cell line could be useful for drug discovery and pathological analyses.
[87]	2020	Developmental Epileptic encephalopathy (DEE), *KCNA2*	Dermal Fibroblasts	3x iPSCs	Patient derived iPSCs	*KCNA2*	The iPSCs with *KCNA2* gene mutation were created from the skin fibroblasts of patients and the pluripotency of the generated cells was confirmed. This cell line could be useful for drug discovery and pathological analyses.
[88]	2020	Epileptic encephalopathy with electrical status epilepticus during sleep (ESES)	PBMCs	iPSCs	Patient derived iPSCs	*KCNA2*	The iPSCs with *KCNA2* gene mutation were created from the PBMCs of the patient and the pluripotency of the generated cells was confirmed. This cell line could be useful for drug discovery and pathological analyses.
[89]	2020	Progressive myoclonic epilepsy (PME)/ myoclonus Epilepsy and Ataxia due to Potassium channel mutation (MEAK)	PBMCs	iPSCs	Patient derived iPSCs	*KCNC1*	The iPSCs with *KCNA1* gene mutation were created from the PBMCs of the patient and the pluripotency of the generated cells was confirmed. This cell line could be useful for drug discovery and pathological analyses.

iPSCs (induced pluripotent stem cells); ESCs (embryonic stem cells), PBMCs (peripheral blood mononuclear cells); NPCs (neuronal progenitors); MGE (medial ganglionic eminence); CGE (caudal ganglionic eminence); NSCs (neuronal stem cells); MEA (microarray electrode analysis)

References

1. Higurashi, N., et al., *A human Dravet syndrome model from patient induced pluripotent stem cells.* Molecular brain, 2013. **6**(1): p. 19.
2. Liu, J., et al., *CRISPR/Cas9 facilitates investigation of neural circuit disease using human iPSCs: mechanism of epilepsy caused by an SCN1A loss-of-function mutation.* Translational psychiatry, 2016. **6**(1): p. e703.
3. Kim, H.W., et al., *Differential effects on sodium current impairments by distinct SCN1A mutations in GABAergic neurons derived from Dravet syndrome patients.* Brain and Development, 2018. **40**(4): p. 287-298.
4. Liu, Y., et al., *Dravet syndrome patient‐derived neurons suggest a novel epilepsy mechanism.* Annals of neurology, 2013. **74**(1): p. 128-139.
5. Maeda, H., et al., *Establishment of isogenic iPSCs from an individual with SCN1A mutation mosaicism as a model for investigating neurocognitive impairment in Dravet syndrome.* Journal of human genetics, 2016. **61**(6): p. 565.
6. Chen, W., et al., *Generation of the SCN1A epilepsy mutation in hiPS cells using the TALEN technique.* Scientific reports, 2014. **4**: p. 5404.
7. Sun, Y. and R.E. Dolmetsch. *Investigating the therapeutic mechanism of cannabidiol in a human induced pluripotent stem cell (iPSC)-based model of Dravet syndrome*. in *Cold Spring Harbor Symposia on Quantitative Biology*. 2018. Cold Spring Harbor Laboratory Press.
8. Xie, Y., et al., *Comparisons of dual isogenic human iPSC pairs identify functional alterations directly caused by an epilepsy associated SCN1A mutation.* Neurobiology of Disease, 2020. **134**: p. 104627.
9. Jiao, J., et al., *Modeling Dravet syndrome using induced pluripotent stem cells (iPSCs) and directly converted neurons.* Hum Mol Genet, 2013. **22**(21): p. 4241-52.
10. Schuster, J., et al., *Generation of three human induced pluripotent stem cell (iPSC) lines from three patients with Dravet syndrome carrying distinct SCN1A gene mutations.* Stem Cell Research, 2019. **39**: p. 101523.
11. Lu, C., et al., *Overexpression of NEUROG2 and NEUROG1 in human embryonic stem cells produces a network of excitatory and inhibitory neurons.* Faseb Journal, 2019. **33**(4): p. 5287-5299.
12. Fruscione, F., et al., *PRRT2 controls neuronal excitability by negatively modulating Na+ channel 1.2/1.6 activity.* Brain, 2018. **141**(4): p. 1000-1016.
13. Martin, P., et al., *TSC patient-derived isogenic neural progenitor cells reveal altered early neurodevelopmental phenotypes and rapamycin-induced MNK-eIF4E signaling.* Molecular autism, 2020. **11**(1): p. 1-15.
14. Rocktäschel, P., A. Sen, and M.Z. Cader, *High glucose concentrations mask cellular phenotypes in a stem cell model of tuberous sclerosis complex.* Epilepsy and Behavior, 2019. **101**.
15. Winden, K.D., et al., *Biallelic mutations in TSC2 lead to abnormalities associated with cortical tubers in human iPSC-derived neurons.* Journal of Neuroscience, 2019. **39**(47): p. 9294-9305.
16. Costa, V., et al., *mTORC1 inhibition corrects neurodevelopmental and synaptic alterations in a human stem cell model of tuberous sclerosis.* Cell reports, 2016. **15**(1): p. 86-95.
17. Zucco, A.J., et al., *Neural progenitors derived from tuberous sclerosis complex patients exhibit attenuated PI3K/AKT signaling and delayed neuronal differentiation.* Molecular and Cellular Neuroscience, 2018. **92**: p. 149-163.

18. Alsaqati, M., V.M. Heine, and A.J. Harwood, *Pharmacological intervention to restore connectivity deficits of neuronal networks derived from ASD patient iPSC with a TSC2 mutation.* Molecular autism, 2020. **11**(1): p. 1-13.
19. Fink, J.J., et al., *Disrupted neuronal maturation in Angelman syndrome-derived induced pluripotent stem cells.* Nature communications, 2017. **8**: p. 15038.
20. Bershteyn, M., et al., *Human iPSC-derived cerebral organoids model cellular features of lissencephaly and reveal prolonged mitosis of outer radial glia.* Cell stem cell, 2017. **20**(4): p. 435-449. e4.
21. Chou, S.-J., et al., *Impaired ROS scavenging system in human induced pluripotent stem cells generated from patients with MERRF syndrome.* Scientific reports, 2016. **6**: p. 23661.
22. Guemez - Gamboa, A., et al., *Loss of Protocadherin - 12 L eads to D iencephalic - M esencephalic J unction D ysplasia S yndrome.* Annals of neurology, 2018. **84**(5): p. 638-647.
23. Zhu, W., et al., *Precisely controlling endogenous protein dosage in hPSCs and derivatives to model FOXG1 syndrome.* Nature Communications, 2019. **10**(1).
24. Homan, C.C., et al., *PCDH19 regulation of neural progenitor cell differentiation suggests asynchrony of neurogenesis as a mechanism contributing to PCDH19 Girls Clustering Epilepsy.* Neurobiology of Disease, 2018. **116**: p. 106-119.
25. Shahsavani, M., et al., *An in vitro model of lissencephaly: expanding the role of DCX during neurogenesis.* Molecular Psychiatry, 2018. **23**(7): p. 1674-1684.
26. Marinowic, D.R., et al., *Induced pluripotent stem cells from patients with focal cortical dysplasia and refractory epilepsy.* Molecular medicine reports, 2017. **15**(4): p. 2049-2056.
27. Majolo, F., et al., *Notch signaling in human iPS-derived neuronal progenitor lines from Focal Cortical Dysplasia patients.* International Journal of Developmental Neuroscience, 2018. **69**: p. 112-118.
28. Majolo, F., et al., *Migration and synaptic aspects of neurons derived from human induced pluripotent stem cells from patients with focal cortical dysplasia II.* Neuroscience, 2019. **408**: p. 81-90.
29. Marinowic, D.R., et al., *Analysis of genes involved in cell proliferation, adhesion, and control of apoptosis during embryonic neurogenesis in Induced Pluripotent Stem Cells (iPSCs) from patients with Focal Cortical Dysplasia.* Brain Research Bulletin, 2020. **155**: p. 112-118.
30. Klofas, L.K., et al., *DEPDC5 haploinsufficiency drives increased mTORC1 signaling and abnormal morphology in human iPSC-derived cortical neurons.* Neurobiology of disease, 2020. **143**: p. 104975.
31. Pelkonen, A., et al., *A modular brain-on-a-chip for modelling epileptic seizures with functionally connected human neuronal networks.* Biosensors and Bioelectronics, 2020. **168 (no pagination)**(112553).
32. Burnight, E.R., et al., *CRISPR-Cas9-Mediated correction of the 1.02 kb common deletion in CLN3 in induced pluripotent stem cells from patients with batten disease.* The CRISPR journal, 2018. **1**(1): p. 75-87.
33. Germain, N.D., et al., *Gene expression analysis of human induced pluripotent stem cell-derived neurons carrying copy number variants of chromosome 15q11-q13. 1.* Molecular autism, 2014. **5**(1): p. 44.
34. Gillentine, M.A., et al., *Functional consequences of CHRNA7 copy-number alterations in induced pluripotent stem cells and neural progenitor cells.* The American Journal of Human Genetics, 2017. **101**(6): p. 874-887.
35. Khan, T. and S. Pasca, *neuronal Defects in a Human cellular Model of 22q11.2 Deletion Syndrome.* Gene Expression Omnibus, 2020.
36. Chia, P.H., et al., *A homozygous loss-of-function camk2a mutation causes growth delay, frequent seizures and severe intellectual disability.* eLife, 2018. **7**.

37. Patzke, C. and T.C. Südhof, *The conditional KO approach: Cre/Lox technology in human neurons.* Rare Diseases, 2016. **4**(1): p. 3560-; 3571.
38. Yamashita, S., et al., *Mislocalization of syntaxin-1 and impaired neurite growth observed in a human iPSC model for STXBP1-related epileptic encephalopathy.* Epilepsia, 2016. **57**(4): p. e81-e86.
39. Perenthaler, E., et al., *Loss of UGP2 in brain leads to a severe epileptic encephalopathy, emphasizing that bi-allelic isoform-specific start-loss mutations of essential genes can cause genetic diseases.* Acta Neuropathol, 2020. **139**(3): p. 415-442.
40. Quraishi, I.H., et al., *An epilepsy-associated KCNT1 mutation enhances excitability of human iPSC-derived neurons by increasing slack K<inf>Na</inf> currents.* Journal of Neuroscience, 2019. **39**(37): p. 7438-7449.
41. van Diepen, L., et al., *A patient-specific induced pluripotent stem cell model for West syndrome caused by ST3GAL3 deficiency.* Eur J Hum Genet, 2018. **26**(12): p. 1773-1783.
42. Tidball, A.M., et al., *Variant-specific changes in persistent or resurgent sodium current in SCN8A-related epilepsy patient-derived neurons.* Brain, 2020. **143**(10): p. 3025-3040.
43. Dang, L.T., et al., *Multimodal Analysis of STRADA Function in Brain Development.* Front Cell Neurosci, 2020. **14**: p. 122.
44. Simkin, D., et al., *Dyshomeostatic modulation of Ca2+-activated K+ channels in a human neuronal model of KCNQ2 encephalopathy.* Elife, 2021. **10**: p. e64434.
45. Glaß, H., et al., *Combined Dendritic and Axonal Deterioration Are Responsible for Motoneuronopathy in Patient-Derived Neuronal Cell Models of Chorea-Acanthocytosis.* Int J Mol Sci, 2020. **21**(5).
46. Gunnewiek, T.M.K., et al., *m.3243A > G-Induced Mitochondrial Dysfunction Impairs Human Neuronal Development and Reduces Neuronal Network Activity and Synchronicity.* Cell Rep, 2020. **31**(3).
47. Marchetto, M.C., et al., *A model for neural development and treatment of Rett syndrome using human induced pluripotent stem cells.* Cell, 2010. **143**(4): p. 527-539.
48. Ricciardi, S., et al., *CDKL5 ensures excitatory synapse stability by reinforcing NGL-1–PSD95 interaction in the postsynaptic compartment and is impaired in patient iPSC-derived neurons.* Nature cell biology, 2012. **14**(9): p. 911-923.
49. Di Matteo, F., et al., *Cystatin B is essential for proliferation and interneuron migration in individuals with EPM1 epilepsy.* EMBO Mol Med, 2020. **12**(6).
50. Fatima, A., et al., *Generation of a human Neurochondrin deficient iPSC line KICRi002-A-3 using CRISPR/Cas9.* Stem Cell Res, 2020. **44**.
51. Meganathan, K., et al., *Regulatory networks specifying cortical interneurons from human embryonic stem cells reveal roles for CHD2 in interneuron development.* Proc Natl Acad Sci U S A, 2017. **114**(52): p. E11180-E11189.
52. Nebel, R., et al., *Reduced CYFIP1 in iPSC Derived Human Neural Progenitors Results in Donor Specific Dysregulation of Schizophrenia and Epilepsy Genes.* Neuropsychopharmacology, 2015. **40**: p. S379-S379.
53. Poppe, D., et al., *Genome Editing in Neuroepithelial Stem Cells to Generate Human Neurons with High Adenosine‐Releasing Capacity.* Stem cells translational medicine, 2018. **7**(6): p. 477-486.
54. da Costa, R.F.M., et al., *Valproate Disturbs Morphology and Mitochondrial Membrane Potential in Human Neural Cells.* Applied In Vitro Toxicology, 2015. **1**(4): p. 254-261.
55. Ahn, S., et al., *Differentiation of human pluripotent stem cells into Medial Ganglionic Eminence vs. Caudal Ganglionic Eminence cells.* Methods, 2016. **101**: p. 103-112.

56. Ishii, M.N., et al., *Human induced pluripotent stem cell (hiPSC)-derived neurons respond to convulsant drugs when co-cultured with hiPSC-derived astrocytes.* Toxicology, 2017. **389**: p. 130-138.
57. Nagel, D.A., et al., *Effects of Lithium and Valproic Acid on Gene Expression and Phenotypic Markers in an NT2 Neurosphere Model of Neural Development.* Figshare, 2013. **1**.
58. Kardoost, M., et al., *Genotoxicity assessment of antiepileptic drugs (AEDs) in human embryonic stem cells.* Epilepsy research, 2019. **158**: p. 106232.
59. Nicholas, C.R., et al., *Functional Maturation of hPSC-Derived Forebrain Interneurons Requires an Extended Timeline and Mimics Human Neural Development.* Cell Stem Cell, 2013. **12**(5): p. 573-586.
60. Bell, S., et al., *Differentiation of Human Induced Pluripotent Stem Cells (iPSCs) into an Effective Model of Forebrain Neural Progenitor Cells and Mature Neurons.* Bio Protoc, 2019. **9**(5): p. e3188.
61. Schwarz, N., et al., *Establishment of a human induced pluripotent stem cell (iPSC)line (HIHDNEi002-A)from a patient with developmental and epileptic encephalopathy carrying a KCNA2 (p.Arg297Gln)mutation.* Stem Cell Res, 2019. **37 (no pagination)**(101445).
62. Germain, N.D., et al., *Derivation and isolation of NKX2.1-positive basal forebrain progenitors from human embryonic stem cells.* Stem Cells Dev, 2013. **22**(10): p. 1477-89.
63. Odawara, A., et al., *Physiological maturation and drug responses of human induced pluripotent stem cell-derived cortical neuronal networks in long-term culture.* Sci Rep, 2016. **6**: p. 26181.
64. Tukker, A.M., et al., *Towards animal-free neurotoxicity screening: Applicability of hiPSC-derived neuronal models for in vitro seizure liability assessment.* Altex, 2020. **37**(1): p. 121-135.
65. Vazin, T., et al., *The effect of multivalent Sonic hedgehog on differentiation of human embryonic stem cells into dopaminergic and GABAergic neurons.* Biomaterials, 2014. **35**(3): p. 941-948.
66. DeRosa, B.A., et al., *hVGAT-mCherry: A novel molecular tool for analysis of GABAergic neurons derived from human pluripotent stem cells.* Mol Cell Neurosci, 2015. **68**: p. 244-57.
67. Giacomoni, J., et al., *Direct conversion of human stem cell-derived glial progenitor cells into GABAergic interneurons.* Cells, 2020. **9**(11): p. 1-17.
68. Hartley, B.J., et al., *In vitro Differentiation of Pluripotent Stem Cells towards either Forebrain GABAergic or Midbrain Dopaminergic Neurons*. 2015. 91-99.
69. Inglis, G.A.S., et al., *Transcriptomic and epigenomic dynamics associated with development of human iPSC-derived GABAergic interneurons.* Hum Mol Genet, 2020. **29**(15): p. 2579-2595.
70. Kreir, M., et al., *Role of Kv7.2/Kv7.3 and M(1) muscarinic receptors in the regulation of neuronal excitability in hiPSC-derived neurons.* Eur J Pharmacol, 2019. **858**: p. 172474.
71. Fedele, D.E., et al., *Engineering embryonic stem cell derived glia for adenosine delivery.* Neurosci Lett, 2004. **370**(2-3): p. 160-165.
72. Chi, L., et al., *Targeted Differentiation of Regional Ventral Neuroprogenitors and Related Neuronal Subtypes from Human Pluripotent Stem Cells.* Stem Cell Reports, 2016. **7**(5): p. 941-954.
73. Ishikawa, M., et al., *miRNA-based rapid differentiation of purified neurons from hPSCs advancestowards quick screening for neuronal disease phenotypes in vitro.* Cells, 2020. **9 (3) (no pagination)**(532).

74. Maroof, A.M., et al., *Directed differentiation and functional maturation of cortical interneurons from human embryonic stem cells.* Cell Stem Cell, 2013. **12**(5): p. 559-72.
75. Miskinyte, G., et al., *Direct conversion of human fibroblasts to functional excitatory cortical neurons integrating into human neural networks.* Stem Cell Res Ther, 2017. **8**(1): p. 207.
76. Tanaka, Y., et al., *Establishment of a human induced stem cell line (FUi002-A) from Dravet syndrome patient carrying heterozygous R1525X mutation in SCN1A gene.* Stem cell research, 2018. **31**: p. 11-15.
77. Tanaka, Y., et al., *Generation of D1-1 TALEN isogenic control cell line from Dravet syndrome patient iPSCs using TALEN-mediated editing of the SCN1A gene.* Stem cell research, 2018. **28**: p. 100-104.
78. Tan, G.W., et al., *Induced pluripotent stem cells derived from an autosomal dominant lateral temporal epilepsy (ADLTE) patient carrying S473L mutation in leucine-rich glioma inactivated 1 (LGI1).* Stem cell research, 2017. **24**: p. 12-15.
79. Schwarz, N., et al., *Generation of an induced pluripotent stem cell (iPSC) line from a patient with developmental and epileptic encephalopathy carrying a KCNA2 (p. Leu328Val) mutation.* Stem cell research, 2018. **33**: p. 6-9.
80. Amenduni, M., et al., *iPS cells to model CDKL5-related disorders.* Eur J Hum Genet, 2011. **19**(12): p. 1246-55.
81. Kimura, Y., et al., *Establishment of human induced pluripotent stem cells derived from skin cells of a patient with Dravet syndrome.* Stem Cell Research, 2020. **47**.
82. Malerba, N., et al., *Generation of the induced human pluripotent stem cell lines CSSi009-A from a patient with a GNB5 pathogenic variant, and CSSi010-A from a CRISPR/Cas9 engineered GNB5 knock-out human cell line.* Stem Cell Research, 2019. **40**.
83. Sun, C., et al., *Generation of an induced pluripotent stem cell line SYSUi-003-A from a child with epilepsy carrying GRIN2A mutation.* Stem Cell Research, 2020. **43**.
84. Zhang, B., et al., *Generation of a human induced pluripotent stem cell line from an epilepsy patient carrying mutations in the PIK3R2 gene.* Stem Cell Research, 2020. **44**.
85. Zhao, H., et al., *Generation of corrected-hiPSC (USTCi001-A-1) from epilepsy patient iPSCs using TALEN-mediated editing of the SCN1A gene.* Stem Cell Research, 2020. **46**.
86. Zhao, H., et al., *Generation of iPSC line (USTCi001-A) from human skin fibroblasts of a patient with epilepsy.* Stem Cell Research, 2020. **45**: p. 101785.
87. Arbini, A., et al., *Generation of three induced pluripotent stem cell (iPSC) lines from a patient with developmental epileptic encephalopathy due to the pathogenic KCNA2 variant c.869T>G; p.Leu290Arg (NUIGi052-A, NUIGi052-B, NUIGi052-C).* Stem Cell Res, 2020. **46 (no pagination)**(101853).
88. Gong, P., et al., *Generation of a human iPSC line from an epileptic encephalopathy patient with electrical status epilepticus during sleep carrying KCNA2 (p.P405L) mutation.* Stem Cell Res, 2020. **49**: p. 102080.
89. Nengqing, L., et al., *Generation of induced pluripotent stem cell GZHMCi001-A and GZHMCi001-B derived from peripheral blood mononuclear cells of epileptic patients with KCNC1 mutation.* Stem Cell Res, 2020. **47 (no pagination)**(101897).
